# Fundamental Definitions for Axially-Strained Piezo-Semiconductive Nanostructures

**DOI:** 10.3390/mi12010020

**Published:** 2020-12-27

**Authors:** Peyman Amiri, Christian Falconi

**Affiliations:** Department of Electronic Engineering, University of Rome Tor Vergata, 00133 Roma, Italy; peymanamiri1992@gmail.com

**Keywords:** piezoelectric nanotransducers, depletion piezopotential, enhancement piezopotential, base piezopotential, tip piezopotential, characteristic lengths of piezopotentials, depletion-to-enhancement piezopotential ratio, tip-to-base piezopotential ratio, piezoelectric nanogenerators, piezotronics

## Abstract

Piezoelectric nanotransducers may offer key advantages in comparison with conventional piezoelectrics, including more choices for types of mechanical input, positions of the contacts, dimensionalities and shapes. However, since most piezoelectric nanostructures are also semiconductive, modeling becomes significantly more intricate and, therefore, the effects of free charges have been considered only in a few studies. Moreover, the available reports are complicated by the absence of proper nomenclature and figures of merit. Besides, some of the previous analyses are incomplete. For instance, the local piezopotential and free charges within axially strained conical piezo-semiconductive nanowires have only been systematically investigated for very low doping (10^16^ cm^−3^) and under compression. Here we give the definitions for the enhancement, depletion, base and tip piezopotentials, their characteristic lengths and both the tip-to-base and the depletion-to-enhancement piezopotential-ratios. As an example, we use these definitions for analyzing the local piezopotential and free charges in n-type ZnO truncated conical nanostructures with different doping levels (intrinsic, 10^16^ cm^−3^, 10^17^ cm^−3^) for both axial compression and traction. The definitions and concepts presented here may offer insight for designing high performance piezosemiconductive nanotransducers.

## 1. Introduction

Piezoelectric nanotransducers [1] may offer key advantages with respect to conventional piezoelectric devices and their potential has already been demonstrated in a wide variety of applications, including strain [2] and acceleration sensing [3], measurement of cellular deformations as small as 1 nm [4], human-motion harvesting [5,6], implantable devices for energy harvesting [7,8,9,10], optoelectronics [11], piezocatalysis [12,13], gas detection [14], temperature sensing [15,16], wireless transducers for converting ultrasounds into electrical stimulations [17,18,19,20], and more. In particular, in comparison with traditional piezoelectric transducers, at nanoscale there are more degrees of freedom as there can be more options for the types of mechanical input [1,21], the positions of the contacts [1,21], the dimensionalities [1,21], and the shapes [1,21,22,23,24]. Despite these and other [1] opportunities, nanoscale dimensions typically result in complications associated to fabricating, characterizing and packaging that make simulations possibly even more important than for conventional devices [25,26]. After the first report on piezoelectric nanogenerators [27], the electric potential within a ZnO nanowire laterally bent by the tip of an atomic force microscope was computed [28]. Subsequently, simulations showed that this configuration is not ideal for energy harvesting because the region under maximum strain is somehow screened by the conductive seed layer [21] and other types of mechanical input, positions of contacts and shapes were systematically investigated [21], including vertical compression which was numerically found to be superior for energy harvesting. Though these simulations offered precious insight for design and led to conclusions confirmed by many experiments, for simplicity, free charges were not taken into account, which is generally a crude approximation as in most cases piezoelectric nanotransducers are made of materials which are both piezoelectric and semiconductive. For instance, in contrast with many other piezoelectric and insulating materials, ZnO nanostructures can be synthesized with low-temperature, low-cost solution methods [29,30] on practically every substrate, including flexible and stretchable substrates or even conventional printed circuit boards [31]. ZnO is a semiconductor and even in absence of intentional doping may not be considered as insulating or even intrinsic as ZnO nanostructures typically exhibit a natural n-type behavior, likely due to non-perfect stoichiometry native defects such as oxygen vacancies or interstitial zinc atoms or hydrogen [32]. As a result, though more simplified simulations can still be useful and provide preliminary information [21,24,28,33], for accurate analyses free charges must be considered [22,23,33,34,35,36]. In fact, cylindrical ZnO nanowires have been simulated with taking into account the presence of free charges both in case of lateral bending [33] and vertical compression [34]. Similarly, the piezopotential in vertically compressed [22,23] or laterally bent [23] truncated conical ZnO nanowires has been studied for different doping levels. However, in these early works, the discussion is complicated by the absence of proper nomenclature and figures of merit. Important cases have not been considered. Here we give fundamental definitions for enabling more accurate and more systematic analyses of axially-strained piezo-semiconductive nanowires. As an important example, we apply these definitions to axially strained truncated conical nanowires. In fact, the local piezopotential and free charges within axially strained conical piezo-semiconductive nanowires have only been investigated for very low doping (10^16^ cm^−3^) and only under compression [22]. In fact, though, at least for the case of compression, the potential difference between the tip and the base has also been computed for different doping levels [23], both the local electrical potential and free charges have not been reported (for both compression and traction). We compute the local piezopotentials as well as the carrier concentrations along the nanostructure both in case of traction and compression for 2 µm long ZnO truncated conical nanowires with different doping levels (intrinsic, 10^16^ cm^−3^ and 10^17^ cm^−3^), base radius equal to 150 nm and radius of the tip ranging from 25 to 125 nm.

## 2. Materials and Methods

**Figure 1:**
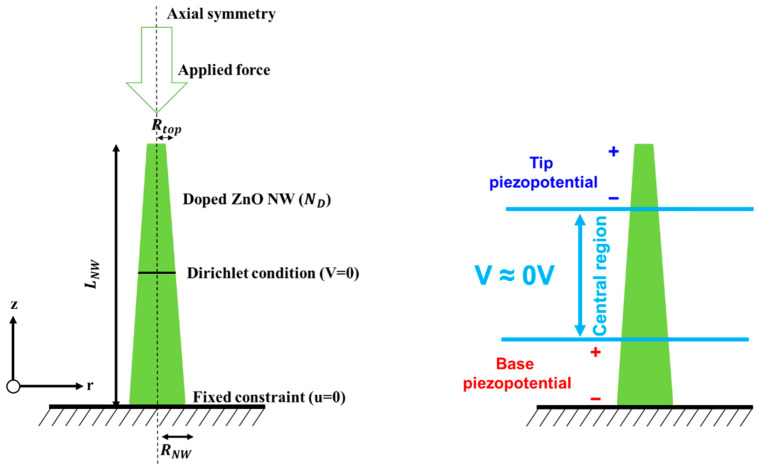
Schematic representations of the truncated conical nanowires illustrating (**left**) the boundary conditions and the coordinate system, with definitions of *L_NW_* (length), *R_NW_* (base radius), *R_TIP_* (tip radius) and (**right**) the tip piezopotential, the base piezopotential and the central region with an almost zero potential.

For simulations, similar to previous studies [22,23,28,33,34,36] we consider ZnO nanostructures because of their remarkable practical advantages, including piezoelectricity and easy fabrication with low-temperature, low-cost, large-area wet-chemical methods which are suitable for almost every substrate, including CMOS devices or plastic and flexible substrates. We used the ZnO conical nanowires and boundary conditions schematically shown in Figure 1. The nanowire length is 2µm and the bottom radius is 150 nm. The tip radius is smaller than the bottom radius in order to form a truncated conical structure and has been changed from 25 to 125 nm. Axial compression or traction forces equal to 442 nN have been uniformly applied [22,34] to the nanowire tip and both the electric potential and the free charges concentrations have been calculated. At the bottom of the nanowire we applied a mechanical constrain (u = 0) and the tapered tip is only free to move axially. At the center of the nanowire the electric potential is fixed to V = 0 in order to not perturb the piezopotential developed at the bottom of the nanowire [22]. The nanowire is surrounded by air. The material properties for ZnO are given in Table 1.

The COMSOL software has been used to simulate the nanowire. The geometry has been created using the 2D axisymmetric method. The zero-displacement condition has been applied to the bottom of the nanowire while the top tip is free to move and will experience either compression or traction force. The Dirichlet condition (V = 0) is applied at the center of the nanowire. In order to consider the effect of charge carriers, the equations for semiconductors have been added to the simulations and, similar to [22,23,34], for simplicity, the effects of external charges [37,38,39] have not been considered. The nanowire is assumed to be uniformly n-type doped, with different doping conditions (intrinsic, 10^16^ cm^−3^, 10^17^ cm^−3^).

## 3. Results

### 3.1. Depletion Piezopotential (ΔV_PZ-DEPL_) and Enhancement Piezopotential (ΔV_PZ-ENHANC_)

The application of strain to a piezo-semiconductive nanostructure will result, in comparison with the initial, unstrained nanostructure, in both enhancement and depletion regions where the free charges will be increased or reduced, respectively [22,23,33,34]. In case of vertical compression or traction applied to axial nanostructures with a constant doping, if, for symmetry, the base is left floating [22], there will be only one depletion region and one enhancement region, at the tip or at the base of the nanowire, depending on the type (p or n) of doping and on the sign of the force (traction or compression) [22,34]. If the length of the nanowire is sufficient, the depletion and the enhancement regions will have no overlaps. Each region will have a sharp limit (tip or base) and an asymptotic limit at the center of the nanowire. In such conditions we define the depletion piezopotential, Δ*V_PZ-DEPL_*, and the enhancement piezopotential, Δ*V_PZ-ENHANC_*, as the potential drops in the depletion and in the enhancement regions, respectively. For consistency with literature, both these potentials must be conventionally taken as the difference between the extremity of the (depletion or enhancement, respectively) region closer to the tip and the extremity closer to the base. With these definitions, the total voltage drop between the tip and the base is the sum of the depletion piezopotential and of the enhancement piezopotential. Since we ignore other effects (e.g., the band bending due to the metal-semiconductor interface) and only consider the piezopotentials due to strain, the depletion piezopotential and the enhancement piezopotential will always be concordant. Though the total potential drop already provides important information, in many cases and, for instance, when designing piezotronic devices, both the depletion piezopotential and the enhancement piezopotential are crucial as the charge transport properties at a certain junction will depend on the correspondent local piezopotential.

### 3.2. Tip Piezopotential (ΔV_PZ-TIP_) and Base Piezopotential (ΔV_PZ-BASE_)

In the analysis of conical nanowires, it is also useful to define the tip piezopotential, Δ*V_PZ-TIP_*, and the base piezopotential, Δ*V_PZ-BASE_*, as the potential drops developed at the tip and base, respectively. Similar to the depletion piezopotential and the enhancement piezopotential, as schematically shown in Figure 1, the tip piezopotential and the base piezopotential will also be conventionally taken as the difference between the tip and the center or between the center and the base, respectively. Clearly, depending on the sign of the axial force and on the type of doping, the base and the tip will be depleted and enhanced, respectively, or vice versa. In cylindrical nanowires, the base and tip have the same radius, but in tapered nanowires the tip area may be much smaller than the base area, which may translate in higher strains which will tend to result in higher piezopotentials at the tip.

### 3.3. Characteristic Lengths of the Piezopotentials (Tip, Base, Depletion or Enhancement Piezopotential)

In the analysis of axially strained nanowires it is useful to quantitatively evaluate the width of the enhancement or depletion regions. However, since the charge concentrations can have complex profiles, instead of considering the charges, we may consider the piezopotentials and define, for each piezopotential (tip, base, depletion or enhancement piezopotential), its characteristic length as the width of the region, starting from the extremity (tip or base) and going toward the center, where most of the correspondent piezopotential drop occurs (e.g., for our simulations we will consider 90% of the piezopotential drop).

### 3.4. Depletion-to-Enhancement Piezopotential Ratio (r_PZ,DEPL-TO-ENHANC_)

Since free charges tend to screen the piezopotential, the depletion piezopotential tends to be higher than the enhancement piezopotential. The superior ability of the depletion region to develop a piezopotential when strained may be quantified by the depletion-to-enhancement piezopotential ratio, *r_PZ,DEPL-TO-ENHANC_*, defined as the ratio between the depletion piezopotential and the enhancement piezopotential. Since both the depletion piezopotential and the enhancement piezopotential are always concord, *r_PZ,DEPL-TO-ENHANC_* is always positive.

### 3.5. Tip-to-Base Piezopotential Ratio (r_PZ,TIP-TO-BASE_)

In cylindrical nanowires the base area and the tip area are identical and, therefore, for a given axial input force, the strain is homogeneous along the entire nanostructure. By contrast, for piezo-semiconductive nanostructures with non-constant cross sections [22,23], for a given input force, different regions are subject to different strains. As a consequence, one region, subject to higher strain, may tend to have a higher magnitude of the piezopotential. The ability of the tip to develop an higher piezopotential may be quantified by the tip-to-base piezopotential ratio, *r_PZ,TIP-TO-BASE_*, defined as the ratio between the tip piezopotential and the base piezopotential. Since the tip piezopotential and the base piezopotential are always concord, *r_PZ,TIP-TO-BASE_* is always positive.

### 3.6. Piezopotential in Truncated Conical Dielectric Nanowires under Vertical Compression or Traction

**Figure 2:**
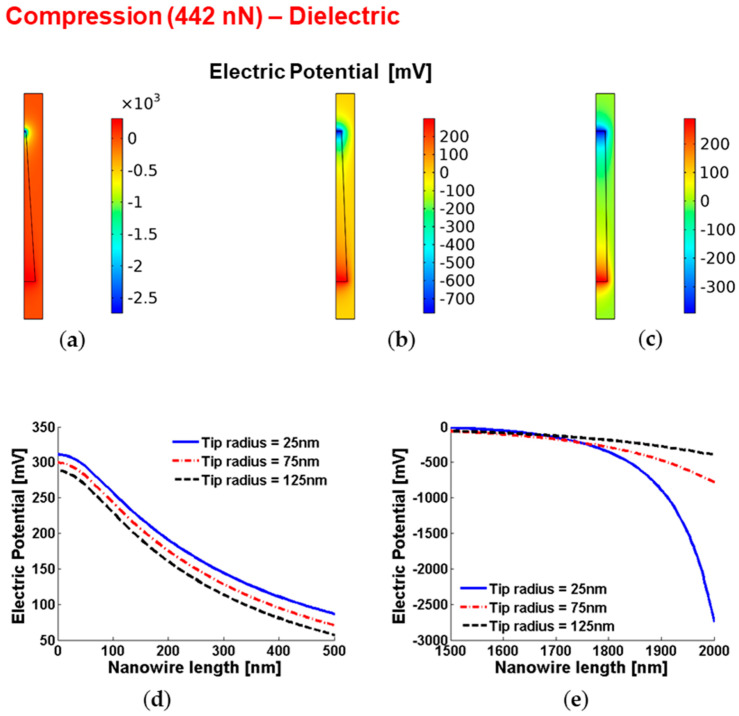
Piezoelectric potential within conical truncated dielectric nanowires with length 2 µm, base radius 150 nm and compressive force 442 nN. (**a**–**c**) Color maps of the piezopotential for tip radius equal to 125 nm (**a**), 75 nm (**b**) and 25 nm (**c**). (**d**,**e**) Base piezopotential (**d**) and tip piezopotential (**e**) for different values of the tip radius.

Figure 2 shows the piezoelectric potential within vertically compressed conical truncated dielectric nanowires. As evident, the local piezopotential at the base of the nanowire is almost unaffected by the change of the tip radius. In fact, for a given compression force, since the base area is constant, the pressure exerted on the base of the nanowire due to the mechanical boundary condition at the base (i.e., no displacement at the base) does not depend on the tip radius. As a consequence, the strain at the base and, therefore, the piezopotential at the base are almost perfectly independent on the tip radius. As expected, when the tip radius is reduced, the higher strain at the tip will result in higher electric field and potential drops.

**Figure 3:**
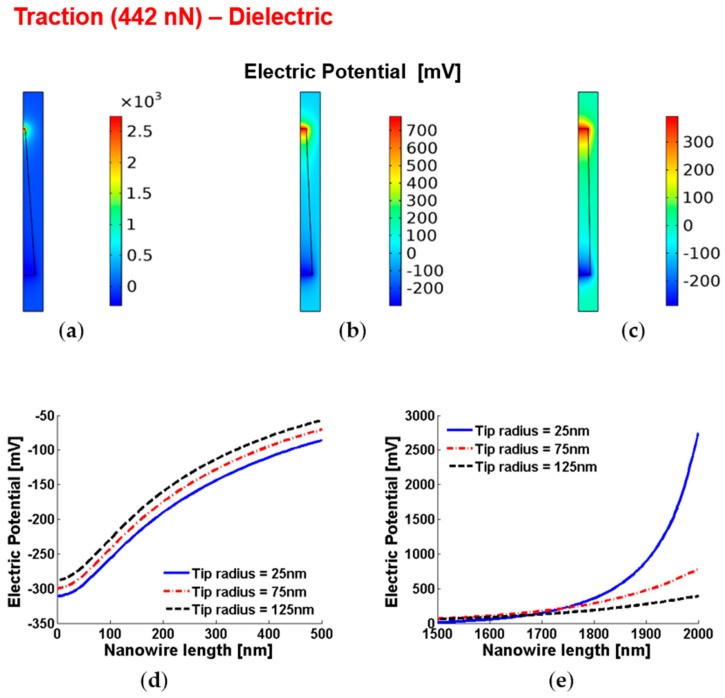
Piezoelectric potential within conical truncated dielectric nanowires with length 2 µm, base radius 150 nm and traction force 442 nN. (**a**–**c**) Color maps of the piezopotential for tip radius equal to 125 nm (**a**), 75 nm (**b**) and 25 nm (**c**). (**d**,**e**) Base piezopotential (**d**) and tip piezopotential (**e**) for different values of the tip radius.

Figure 3 shows similar results for the case of traction. As expected, the sign of the piezopotentials are opposite, but the magnitude of the tip piezopotential is still larger in comparison with the base piezopotential due to the smaller area and, therefore, higher strain at the tip.

### 3.7. Piezopotentials, Free Carrier Concentrations, Piezopotential Ratios and Characteristic Lengths of the Piezopotentials in Truncated Conical Nanowires with 10^16^ cm^−3^ Doping under Vertical Compression or Traction

**Figure 4:**
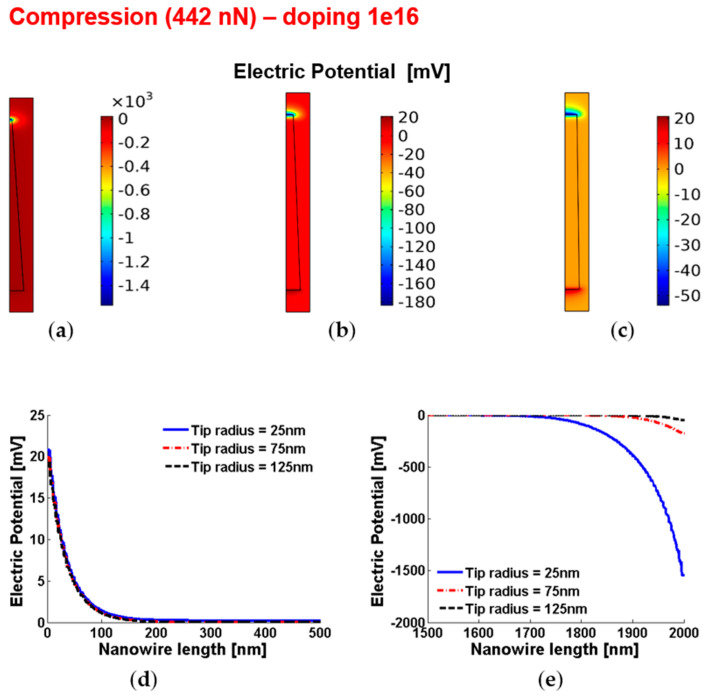
Piezoelectric potential within conical truncated nanowires with length 2 µm, base radius 150 nm, compressive force 442 nN and n-type 10^16^ cm^−3^ doping. (**a**–**c**) Color maps of the piezopotential for tip radius equal to 125 nm (**a**), 75 nm (**b**) and 25 nm (**c**). (**d**,**e**) Base (enhancement) piezopotential (**d**) and tip (depletion) piezopotential (**e**) for different values of the tip radius.

**Figure 5:**
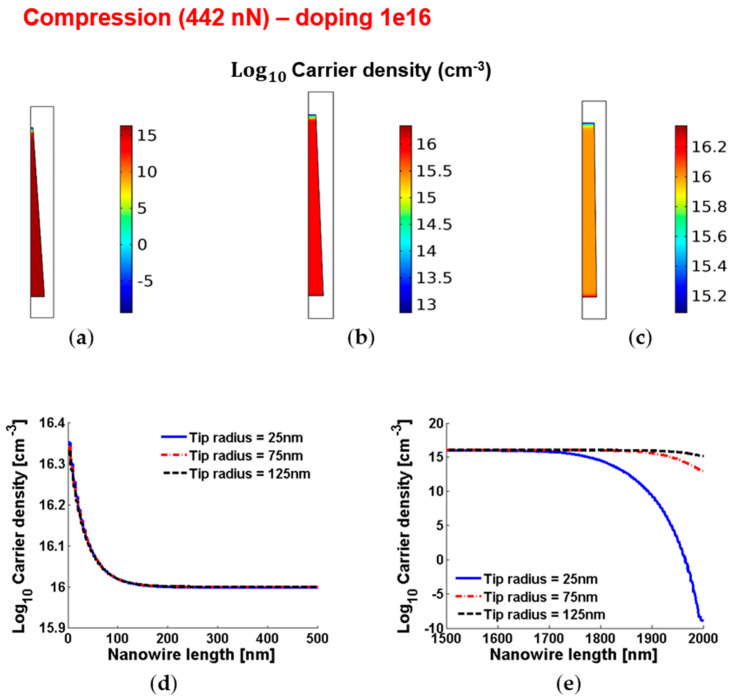
Free charge concentrations within conical truncated nanowires with length 2 µm, base radius 150 nm, compressive force 442 nN and n-type 10^16^ cm^−3^ doping. (**a**–**c**) Color maps of the free charge concentrations for tip radius equal to 125 nm (**a**), 75 nm (**b**) and 25 nm (**c**). (**d**,**e**) Charge concentration at the base (enhancement) (**d**) and tip (depletion) (**e**) for different values of the tip radius.

Figure 4 and Figure 5 show the piezoelectric potential and the free carrier concentrations, respectively, within vertically compressed ZnO truncated conical nanowires with 10^16^ cm^−3^ doping.

**Figure 6:**
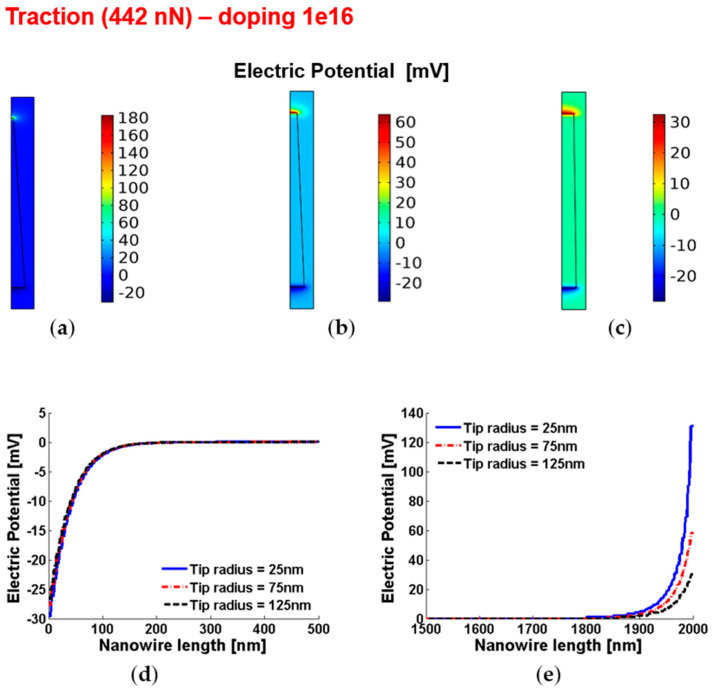
Piezoelectric potential within conical truncated nanowires with length 2 µm, base radius 150 nm, traction force 442 nN and n-type 10^16^ cm^−3^ doping. (**a**–**c**) Color maps of the piezopotential for tip radius equal to 125 nm (**a**), 75 nm (**b**) and 25 nm (**c**). (**d**,**e**) Base (enhancement) piezopotential (**d**) and tip (depletion) piezopotential (**e**) for different values of the tip radius.

**Figure 7:**
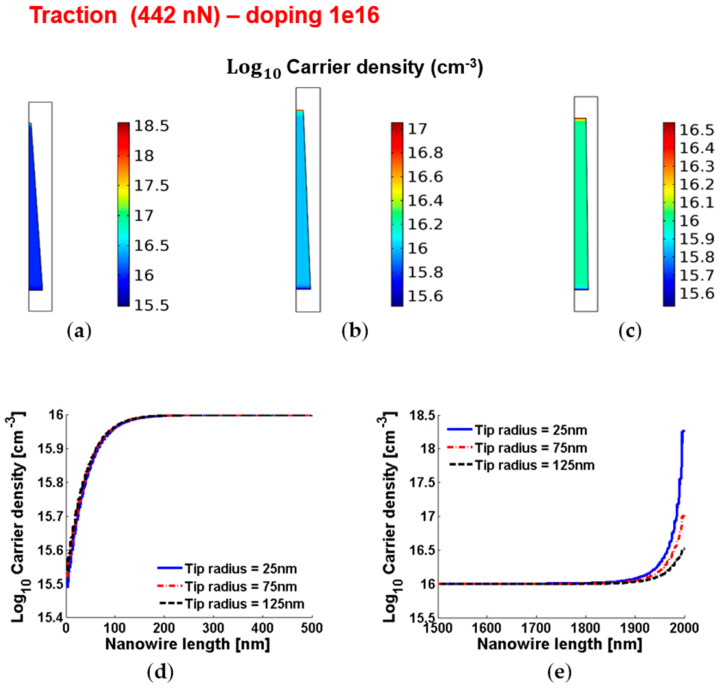
Free charge concentrations within conical truncated nanowires with length 2 µm, base radius 150 nm, traction force 442 nN and n-type 10^16^ cm^−3^ doping. (**a**–**c**) Color maps of the free charge concentrations for tip radius equal to 125 nm (**a**), 75 nm (**b**) and 25 nm (**c**). (**d**,**e**) Charge concentration at the base (enhancement) (**d**) and tip (depletion) (**e**) for different values of the tip radius.

Figure 6 and Figure 7 show the piezoelectric potential and the free carrier concentrations, respectively, within vertically pulled ZnO truncated conical nanowires with 10^16^ cm^−3^ doping.

Table 2 and Table 3 show the piezopotentials, the piezopotentials-ratios and the characteristic lengths of the piezopotentials for 2 µm long n-type ZnO truncated conical nanowires with 10^16^ cm^−3^ doping under axial compression (Table 2) and traction (Table 3).

### 3.8. Piezopotentials, Free Carrier Concentrations, Piezopotential Ratios and Characteristic Lengths of the Piezopotentials in Truncated Conical Nanowires with 10^17^ cm^−3^ Doping under Vertical Compression or Traction

**Figure 8:**
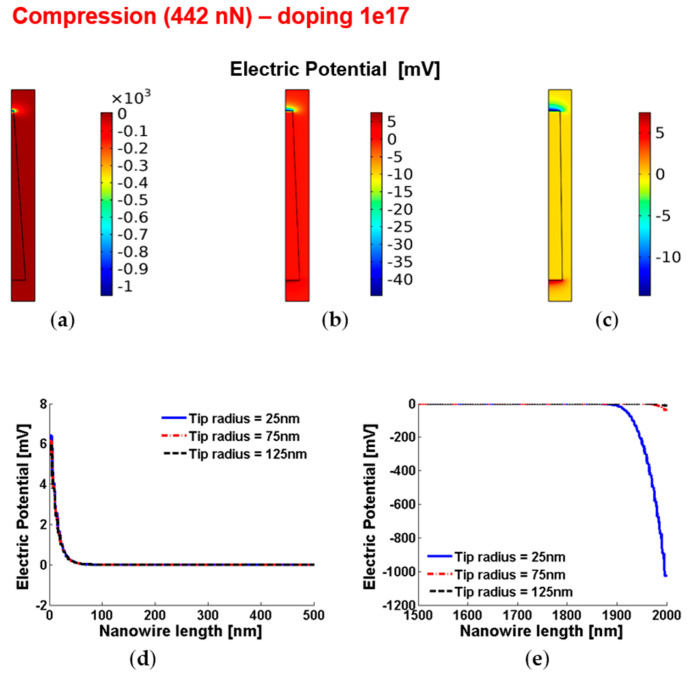
Piezoelectric potential within conical truncated nanowires with length 2 µm, base radius 150 nm, compressive force 442 nN and n-type 10^17^ cm^−3^ doping. (**a**–**c**) Color maps of the piezopotential for tip radius equal to 125 nm (**a**), 75 nm (**b**) and 25 nm (**c**). (**d**,**e**) Base (enhancement) piezopotential (**d**) and tip (depletion) piezopotential (**e**) for different values of the tip radius.

**Figure 9:**
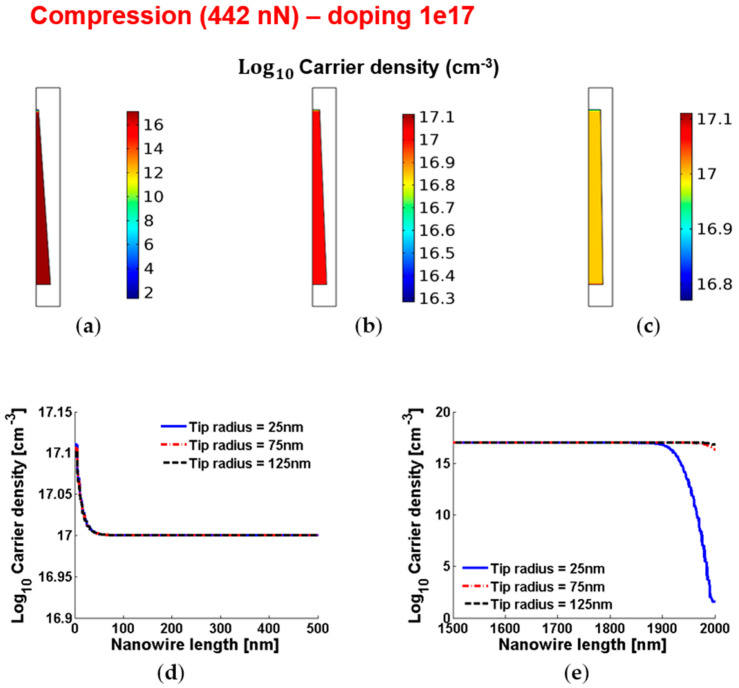
Free charge concentrations within conical truncated nanowires with length 2 µm, base radius 150 nm, compressive force 442 nN and n-type 10^17^ cm^−3^ doping. (**a**–**c**) Color maps of the free charge concentrations for tip radius equal to 125 nm (**a**), 75 nm (**b**) and 25 nm (**c**). (**d**,**e**) Charge concentration at the base (enhancement) (**d**) and tip (depletion) (**e**) for different values of the tip radius.

Figure 8 and Figure 9 show the piezoelectric potential and the free carrier concentrations, respectively, within vertically compressed ZnO truncated conical nanowires with 10^17^ cm^−3^ doping.

**Figure 10:**
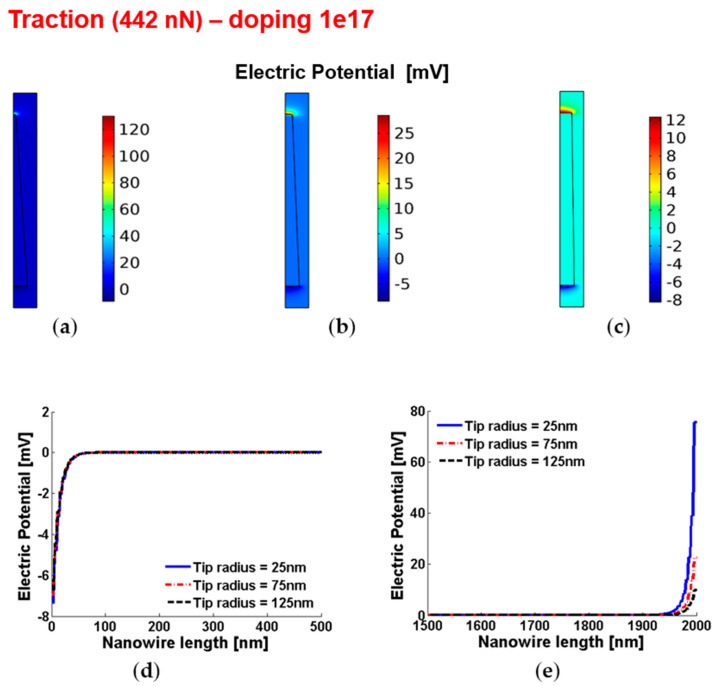
Piezoelectric potential within conical truncated nanowires with length 2 µm, base radius 150 nm, traction force 442 nN and n-type 10^17^ cm^−3^ doping. (**a**–**c**) Color maps of the piezopotential for tip radius equal to 125 nm (**a**), 75 nm (**b**) and 25 nm (**c**). (**d**,**e**) Base (enhancement) piezopotential (**d**) and tip (depletion) piezopotential (**e**) for different values of the tip radius.

**Figure 11:**
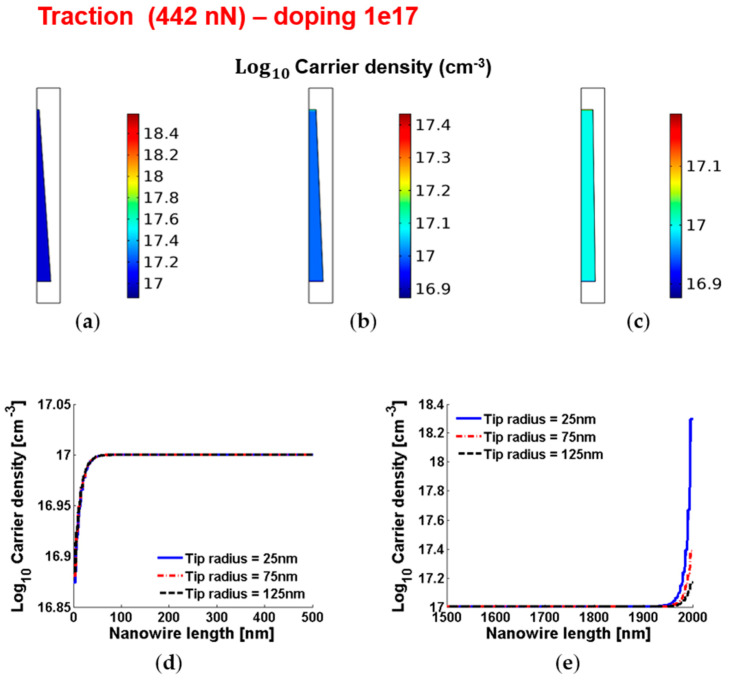
Free charge concentrations within conical truncated nanowires with length 2 µm, base radius 150 nm, traction force 442 nN and n-type 10^17^ cm^−3^ doping. (**a**–**c**) Color maps of the free charge concentrations for tip radius equal to 125 nm (**a**), 75 nm (**b**) and 25 nm (**c**). (**d**,**e**) Charge concentration at the base (enhancement) (**d**) and tip (depletion) (**e**) for different values of the tip radius.

Figure 10 and Figure 11 show the piezoelectric potential and the free carrier concentrations, respectively, within vertically pulled ZnO truncated conical nanowires with 10^17^ cm^−3^ doping.

Table 4 and Table 5 show the piezopotentials, the piezopotentials-ratios and the characteristic lengths of the piezopotentials for 2 µm long n-type ZnO truncated conical nanowires with 10^17^ cm^−3^ doping under axial compression (Table 4) and traction (Table 5).

## 4. Discussion and Conclusions

We have given the fundamental definitions for the analysis of piezosemiconductive nanostructures under axial strain. First, we have defined the depletion piezopotential (Δ*V_PZ-DEPL_*) and the enhancement piezopotential (Δ*V_PZ-ENHANC_*), the tip piezopotential (Δ*V_PZ-TIP_*) and the base piezopotential (Δ*V_PZ-BASE_*). Depending on the type (p or n) of doping and on the sign of the force (traction or compression) [22,34] the tip (base) is enhanced (depleted) or depleted (enhanced) and vice versa. All these piezopotentials can be conventionally taken with their positive terminal closer to the tip (i.e., coincident with the tip if the tip is within the region where the piezopotential is or, otherwise, with the center) and the negative terminal closer to the base (i.e., coincident with the base if the base is within the region where the piezopotential is or, otherwise, with the center). These distinct piezopotentials can only be defined if the nanostructure is sufficiently long so that there is region in the center where the piezopotential is almost perfectly constant (i.e., there is almost zero electric field) and the depletion and enhancement regions are clearly distinguishable at the extremities of the nanowires. If the length of the nanowire is insufficient, the depletion and the enhancement regions will overlap and these piezopotentials may not be defined. Clearly, these definitions only apply to semiconductive nanostructures as in dielectric nanowires there is no depletion or enhancement so that it is not possible to define these piezopotentials.

Afterwards, we have proposed to quantitatively evaluate the widths of the regions where the piezopotential develops, instead of referring to the concentrations of free charges, whose total variations strongly depend on the particular case under investigation (e.g., depend on doping level, geometry and applied force), thus not allowing to easily define a figure of merit. With this simplification, for each piezopotential (tip, base, depletion or enhancement piezopotential), its characteristic length can be defined as the width of the region where most of the correspondent piezopotential drop occurs (e.g., for our simulations we will consider 90% of the piezopotential drop), starting from the extremities (tip or base) and going toward the center.

Other figures of merit which can allow to concisely characterize axially strained piezoelectric nanostructures are the depletion-to-enhancement piezopotential ratio (*r_PZ,DEPL-TO-ENHANC_*) and the tip-to-base piezopotential ratio (*r_PZ,TIP-TO-BASE_*) which quantitatively express the asymmetrical ability of the nanostructure to develop the piezopotential in different regions. With our definitions of the fundamental piezopotentials, both these ratios are always positive. As to the expected values for these ratios, the piezopotential is more easily created in depleted regions (rather than in enhanced regions, because of the reduced number of free charges) and at the tip (rather than at the base, because of the reduced area and, therefore, of the higher strains). As a result, when the tip is depleted, these ratios will be identical and will tend to be very high (much higher than 1), whereas if the tip is enhanced these ratios will be reciprocal (multiplicative inverse) and may be larger or smaller than 1.

Finally, as an example, we have applied these definitions to axially strained truncated conical nanowires and computed the local piezopotential and free charges within axially strained conical piezo-semiconductive nanowires both in case of traction and compression for 2 µm long ZnO truncated conical nanowires with different doping levels (insulator, 10^16^ cm^−3^ and 10^17^ cm^−3^), base radius equal to 150 nm and radius of the tip ranging from 25 nm to 125 nm. In these simulations, as expected [22,23,34], the piezopotential tend to be higher in the depletion region and at the tip so that when the depletion region is at the tip (i.e., the enhancement region is at the base), as it is the case for n-type ZnO nanowires under compression, the ratios *r_PZ,DEPL-TO-ENHANC_* and *r_PZ,TIP-TO-BASE_* are identical and tend to be very high (up to 170), with the highest values found when the tip radius is minimum (i.e., higher pressure and strains at the tip). By contrast, if the depletion region is at the base and the enhancement region is at the tip, as it is the case for n-type ZnO nanowires under traction, the higher strains at the tip do not necessarily result in significantly higher piezopotentials because of the higher concentrations of free charges at the tip. The characteristic lengths of the piezopotentials are always of the same order of magnitude, though, of course, when the depletion region is at the tip and, therefore, under higher strains, the characteristic length of the tip (or depletion) piezopotential may extend significantly more (e.g., up to about 2 times) more than the characteristic length of the base (or enhancement) piezopotential.

Most remarkably, earlier works restricted, at least for some cases (i.e., for some doping level) to general and global quantities, such as the total “tip-to-base” piezopotential drop developed within the nanostructure. Though such an approach already allows to obtain very important information, the charge transport at each junction is controlled by the local piezopotential developed in close proximity of the metal-nanostructure junction and, for this reason, local analyses and local figures of merit, such as those proposed here, are mandatory. Moreover, the figures of merit introduced here allows to easily obtain quantitative insight; for instance, our results show that when doping is increased (from 10^16^ cm^−3^ to 10^17^ cm^−3^), for low tip radius the depletion piezopotential is very well preserved, thus resulting in an even increased depletion-to-enhancement piezopotential ratio. This work can great facilitate systematic analyses of axially strained piezosemiconductive nanostructures and can provide insight for the design of better piezoelectric nanotransducers.

## Figures and Tables

**Table 1:** Properties of the ZnO nanowires used in the simulations.

Young’s Modulus	Poisson’s Ratio	Piezoelectric Coefficients (e33)
129 GPa	v = 0.349	1.22 C m^−2^

**Table 2:** Piezopotentials, piezopotentials-ratios and characteristic lengths of the piezopotentials for 2 µm long n-type ZnO truncated conical nanowires with 150 nm base radius and 10^16^ cm^−3^ doping under axial compression.

*N_D_* = 10^16^ cm^−3^, Compression (442 nN)			
*R_TIP_*	25 nm	75 nm	125 nm
Δ*V_PZ-DEPL_ =* Δ*V_PZ-TIP_*	−1540 mV	−180 mV	−52 mV
Δ*V_PZ-ENHANC_* = Δ*V_PZ-BASE_*	−21 mV	−20 mV	−19 mV
*r_PZ,DEPL-TO-ENHANC_* = *r_PZ,TIP-TO-BASE_*	73	9	2.7
*Characteristic length* (Δ*V_PZ-DEPL_* = Δ*V_PZ-TIP_*)	165 nm	115 nm	94 nm
*Characteristic length* (Δ*V_PZ-ENHANC_* = Δ*V_PZ-BASE_*)	83 nm	80 nm	80 nm

**Table 3:** Piezopotentials, piezopotentials-ratios and characteristic lengths of the piezopotentials for 2 µm long n-type ZnO truncated conical nanowires with 150 nm base radius and 10^16^ cm^−3^ doping under axial traction.

*N_D_* = 10^16^ cm^−3^, Traction (442 nN)			
*R_TIP_*	25 nm	75 nm	125 nm
Δ*V_PZ-DEPL_* = Δ*V_PZ-BASE_*	131 mV	58 mV	30 mV
Δ*V_PZ-ENHANC_* = Δ*V_PZ-TIP_*	30 mV	28 mV	27 mV
*r_PZ,DEPL-TO-ENHANC_* = 1/*r_PZ,TIP-TO-BASE_*	4.4	2.1	1.1
*r_PZ,TIP-TO-BASE_* = 1/*r_PZ,DEPL-TO-ENHANC_*	0.23	0.48	0.9
*Characteristic length* (Δ*V_PZ-ENHANC_* = Δ*V_PZ-TIP_*)	60 nm	74 nm	79 nm
*Characteristic length* (Δ*V_PZ-DEPL_* = Δ*V_PZ-BASE_*)	90 nm	89 nm	89 nm

**Table 4:** Piezopotentials and piezopotentials-ratios for 2 µm long n-type ZnO truncated conical nanowires with 150 nm base radius with 10^17^ cm^−3^ doping under axial compression.

*N_D_* = 10^17^ cm^−3^, Compression (442 nN)			
*R_TIP_*	25 nm	75 nm	125 nm
Δ*V_PZ-DEPL_* = Δ*V_PZ-TIP_*	−1028 mV	−38 mV	−12 mV
Δ*V_PZ-ENHANC_* = Δ*V_PZ-BASE_*	−6 mV	−6 mV	−6 mV
*r_PZ,DEPL-TO-ENHANC_* = *r_PZ,TIP-TO-BASE_*	170	6.3	2
*Characteristic length* (Δ*V_PZ-DEPL_* = Δ*V_PZ-TIP_*)	66 nm	31 nm	30 nm
*Characteristic length* (Δ*V_PZ-ENHANC_* = Δ*V_PZ-BASE_*)	29 nm	29 nm	29 nm

**Table 5:** Piezopotentials, piezopotentials-ratios and characteristic lengths of the piezopotentials for 2 µm long n-type ZnO truncated conical nanowires with 150 nm base radius and 10^17^ cm^−3^ doping under axial traction.

*N_D_* = 10^17^ cm^−3^, Traction (442 nN)			
*R_TIP_*	25 nm	75 nm	125 nm
Δ*V_PZ-DEPL_* = Δ*V_PZ-BASE_*	75 mV	22 mV	10 mV
Δ*V_PZ-ENHANC_* = Δ*V_PZ-TIP_*	7 mV	7 mV	7 mV
*r_PZ,DEPL-TO-ENHANC_* = 1/*r_PZ,TIP-TO-BASE_*	10.7	3.2	1.4
*r_PZ,TIP-TO-BASE_* = 1/*r_PZ,DEPL-TO-ENHANC_*	0.093	0.32	0.7
*Characteristic length* (Δ*V_PZ-ENHANC_* = Δ*V_PZ-TIP_*)	25 nm	26 nm	29 nm
*Characteristic length* (Δ*V_PZ-DEPL_* = Δ*V_PZ-BASE_*)	30 nm	30 nm	30 nm
